# Efficacy of 5 and 10 mg donepezil in improving cognitive function in patients with dementia: a systematic review and meta-analysis

**DOI:** 10.3389/fnins.2024.1398952

**Published:** 2024-07-22

**Authors:** Mehak Sheikh, Mohammad Ammar

**Affiliations:** ^1^Faculty of Pharmaceutical Sciences (FOP), University of Central Punjab, Lahore, Pakistan; ^2^Qatar University Young Scientists Center (QUYSC), Qatar University, Doha, Qatar

**Keywords:** systematic review, meta-analysis, cognition, donepezil, dementia, Alzheimer’s disease, vascular dementia, cognitive impairment

## Abstract

**Objective:**

The purpose of this study was to compare donepezil at 5 mg and 10 mg/day against a placebo to systematically evaluate its effectiveness in improving cognitive function among patients suffering from dementia at any stage.

**Method:**

For this systematic review and meta-analysis, we looked up Medline, Scopus, Embase, Web of Science, and The Cochrane Library for articles on the efficacy of donepezil in dementia published in the past 20 years and summarized the placebo and intervention data. Initially, a total of 2,272 articles were extracted using our search query and after the inclusion and exclusion criteria set for extraction of data, 18 studies were included in this review using PRISMA flowchart. The ADAS-cog and MMSE assessment scales were used for measuring the outcomes using IBM SPSS 29.0 for the meta-analysis.

**Result:**

The meta-analysis comprised a total of 18 RCTs (randomized controlled trials) that were randomized to receive either donepezil 5 mg/day (*n* = 1,556), 10 mg/day (*n* = 2050) or placebo (*n* = 2,342). Meta-analysis concerning efficacy showed that donepezil at 10 mg/day significantly improved the MMSE score (g: 2.27, 95%CI: 1.25–3.29) but could not substantially reduce the ADAS-cog. At 5 mg/day donepezil, an overall slight improvement in MMSE score (Hedges’ g: 2.09, 95%CI: 0.88–3.30) was observed.

**Conclusion:**

Both donepezil 5 mg/day and 10 mg/day doses demonstrated improved cognitive functions for patients with dementia, however results indicated that the 10 mg/day dose was more efficacious.

## 1 Introduction

Dementia is a neurological disorder that usually progresses over time. It is marked by cognitive impairment (language and memory) and non-cognitive alterations (psychosis and behavioral abnormalities; Jin and Liu, 2019; Ohno et al., 2019; Moreno-Morales et al., 2020). According to the World Health Organization (WHO) (WHO, 2023), currently over 55 million individuals are affected by dementia worldwide (WHO, 2023). The most prevalent form of dementia is Alzheimer’s disease (AD; Weller and Budson, 2018; Breijyeh and Karaman, 2020) responsible for about 70% of dementia cases (Aarsland, 2020; WHO, 2023), followed by vascular dementia (VaD; Hu et al., 2022; Morris et al., 2022) constituting about 20% (Aarsland, 2020) of the total cases. Whereas, mild cognitive impairment (MCI) may be considered an early-stage disease in AD as well as VaD (Kasper et al., 2020) with 39.2% of cases progressing to dementia (Bai et al., 2022).

AD is an irreversible and gradual impairment in memory and cognitive functions. It is believed that a decrease in acetylcholine (ACh) production and accumulation of β-amyloid (Aβ) have a major role in its pathogenesis (Cholinesterase inhibitors as Alzheimer’s therapeutics (Review), 2019; Argueta et al., 2022). Unfortunately, AD currently lacks a disease-modifying therapy (DMT; Joe and Ringman, 2019; Noufi et al., 2019; Takramah and Asem, 2022) due to its complex and unclear pathophysiology i.e., genetic or environmental factors (Zhang H, et al., 2022). The success rate of drug development against complete AD progression has been very low and is almost 0% against DMT (Cummings et al., 2019). However, its progression can be slowed and its symptoms can be improved through pharmacotherapy but their therapeutic effects are limited too (Joe and Ringman, 2019; Noufi et al., 2019; Takramah and Asem, 2022). Some studies show that physical exercise can also help improve cognitive symptoms and decrease their rate (Cheng, 2016; Pisani et al., 2021).

Currently, three acetylcholinesterase inhibitors (AChEI; i.e., donepezil, galantamine, and rivastigmine) at any stage of AD and memantine (Argueta et al., 2022) for mild to moderate AD have been authorized by FDA (US Food and Drug Administration) as symptomatic pharmacotherapies (Howard et al., 2012; Grossberg et al., 2013; Tisher and Salardini, 2019). Acetylcholinesterase (AChE) and butyrylcholinesterase (BuChE) are candidate enzymes that are responsible for catabolism of ACh in the brain and therefore, acetylcholinesterase inhibitors (AChEI) are used to increase brain Ach levels by inhibiting these enzymes (Birks and Harvey, 2018; Noufi et al., 2019) as well as inhibit Aβ aggregation (Mezeiova et al., 2019). However, out of the three AChEI, donepezil has shown the most hopeful results and therefore has been the most studied drug (Colwell et al., 2022) approved by the FDA in the late 20th century for mild to moderate AD (Marucci et al., 2021) at doses of 5 mg/day and 10 mg/day. Donepezil is a reversible AChE inhibitor having 1,250 times greater affinity toward AChE than BuChE and an inhibitory effect toward Aβ formation at the same time. It is known to be well-tolerated with a high ability to improve cognitive deficits and global function in AD patients showing minimal side effects (Barfejani et al., 2020; Marucci et al., 2021).

In addition, new monoclonal antibodies against amyloid-β i.e., Bapineuzumab and Solanezumab were introduced for the treatment of AD but their development was terminated by Pfizer and Johnson & Johnson in 2012 (Perng et al., 2018). These drugs failed to execute any better results as compared to placebo in trials conducted at late-stage in patients with mild to moderate AD. One study conducted (Abushouk et al., 2017) for Bapineuzumab and a phase 3 trial study conducted by Eli Lily (Sperling et al., 2023) for Solanezumab confirmed these results. However, aducanumab (BiogenInc), another monoclonal antibody recently got approval from the FDA in 2021 for AD treatment in 20 years (Dunn et al., 2021; Argueta et al., 2022) but is still under investigation concerning its risk–benefit evaluation. Besides this, recent studies have also suggested combination therapies effectively treating cognitive impairment. A very famous combination of donepezil and memantine has shown its effect in improving behavioral and psychological symptoms in dementia (Guo et al., 2020; Rong et al., 2021; Yaghmaei et al., 2024) as well as in moderate to severe AD (Knorz and Quante, 2022a) by activation of glutamatergic neurons. Furthermore, according to the latest studies by (Knorz and Quante, 2022), a neuroprotective agent EGb 761 has been shown to reduce concentrations of amyloid-β and AChE significantly (Rong et al., 2021; Knorz and Quante, 2022). In addition to this, various vitamins (Knorz and Quante, 2022) i.e., 25 OH vitamin D in combination with donepezil (Ware et al., 2016; Rong et al., 2021) have demonstrated a greater response to cognition in the case of AD.

In contrast to AD, currently, there are no approved drugs for the treatment of VaD (Patel and Holland, 2022) and MCI. The progress in the development of effective pharmacotherapy for VaD has been a challenge due to its not so well understood pathogenesis i.e., the relation between cerebrovascular pathology and cognitive impairment (Lagunin et al., 2020). Donepezil 5 and 10 mg/day is currently the most widely used drug for the treatment of cognitive symptoms of VaD (Tisher and Salardini, 2019) and MCI (Chen et al., 2021). However, further study is required to determine its efficacy in the treatment of MCI (Devanand et al., 2018). According to certain studies, there is currently insufficient evidence to warrant using cholinesterase inhibitors to treat MCI (Pisani et al., 2021). Therefore, this meta-analysis lays the groundwork for further investigation on the efficacy of donepezil for the treatment of dementia (mainly AD, VaD, and MCI) in improving cognitive symptoms, comparing the dosages of 5 mg/day and 10 mg/day. To achieve this, the present study addresses the following research questions:

1. What are the most effective assessments to measure cognitive symptoms in dementia?
2. Among 5 mg and 10 mg donepezil doses, which one is more effective in managing cognitive symptoms in patients with dementia, as measured through systematic assessments?

## 2 Methods

For this meta-analysis, a systematic literature review approach was used following the Preferred Reporting Items for Systematic Reviews and Meta-Analyses (PRISMA) guidelines (Page et al., 2021). These guidelines were used because of their ability to review and summarize previous studies while also highlighting areas in which more study is required.

### 2.1 Literature search strategy

To comprehensively cover all the available research, we searched Medline, Scopus, Embase, Web of Science, and The Cochrane Library. These databases were chosen because of their global reputation, offering an ideal blend of resources. We included studies from the past 20 years to make this study timely. Several trial searches were conducted and then in August 2023, the final search was executed. The search terminology followed was: [(“effic*” OR “saf*” OR “outcom*” OR “resul*” OR “effec*” OR “respons*”) AND (“donepezil” OR “aricept”) AND (“alzheime*” OR “dement*” OR “cognitive”) AND (“random* contro*” OR “placebo contro*”)]. The purpose of this search query was to elicit the study’s title, abstract, or keywords. Additionally, these search terms were chosen because of their frequent usage in medical research related to our topic. The search string was simplified which led to the more extensive search, producing the most publications possible. Afterward, these were narrowed down by using article-specific standards.

### 2.2 Inclusion and exclusion criteria

This study included all randomized, double-blind, or placebo-controlled trials randomized either as groups or individuals with both intervention and observation groups. Patients diagnosed with any type of dementia showing cognitive symptoms with no restriction to age, origin, gender, etiology, and cognitive impairment severity were considered research participants. Any intervention that compared donepezil with a placebo in patients with dementia was included in this review. In the treatment group where there were doses other than 5 mg/day or 10 mg/day were also inquired (i.e., 1 mg/day or 3 mg/day) in some studies, only doses of donepezil at 5 mg/day or 10 mg/day and the control group given placebo were considered in present study. Additionally, assessment scales for measurement of cognitive symptoms included in this meta-analysis were the Mini-Mental State Examination Scale (MMSE), and the Alzheimer’s Disease Assessment Scale-Cognitive Subscale (ADAS-cog) at both doses. For the exclusion criteria, the following were considered: (1) Studies that were originally a review, a retrospective study, a case report, or a meta-analysis were excluded; (2) Articles with incomplete, ongoing, or unavailable information were also excluded; and (3) Interventions in which either group was given cholinesterase other than donepezil or any non-pharmacological interventions (i.e., physical or cognitive therapy, music therapy, aromatherapy or physical exercise) were also excluded.

### 2.3 Cognition

Cognitive assessment scales are frequently used in clinical research of geriatric settings to assess the likelihood of dementia (Mahendran et al., 2015). ADAS-Cog and MMSE assessment scales were used in the present study to evaluate the severity and improvement of cognitive impairment in patients.

#### 2.3.1 ADAS-Cog

The purpose of ADAS-Cog is to quantify and track the progression of cognitive symptoms and has been a cornerstone of substantial clinical research and trials since its introduction in the 1980s (Cogo-Moreira et al., 2021, 2023). ADAS is considered more reliable and accurate than MMSE (Kaufman et al., 2023). It mainly evaluates cognitive areas of memory, language, and orientation consisting of 11 tasks scoring of which is done from 0 to 70 as a single scale. Scoring is based on patient-performed tasks and observer-based evaluation and takes about 30–45 min for its completion (Kueper et al., 2018).

#### 2.3.2 MMSE

Mini-Mental State Examination (MMSE) is the most often used screening instrument commonly used for assessing the extent of cognitive impairment in patients and takes about 7–10 min. It has a total of 30 tasks with subgroups covering memory skills, comprehension, reading, writing, and illustrating skills as well as visual construction attention (Saczynski et al., 2015; Chiu et al., 2021; Jia et al., 2021; Su et al., 2021).

### 2.4 Data extraction

Relevant items were retrieved and cross-checked after one independent researcher examined the entirety of the included literature. The gathered data was then examined by a senior researcher. The following data from included trials was added: initial author, eligibility criteria, publishing year, region of research, research design, sample size, clinical group with severity, age, follow-up period, intervention (dose, duration), and methods of outcome assessments. If accessible, we documented the ITT findings.

### 2.5 Statistical methods

The goal of a meta-analysis is to compile the statistical data acquired from several research investigations. We used IBM SPSS 29.0 for statistical analysis of the data. When performing a meta-analysis, there is a trade-off between increasing the number of studies to boost strength as well as narrowing the selection to lower heterogeneity. For this meta-analysis, the raw data retrieved from studies was first inserted into a Microsoft Excel template created according to the design of the present study and was then entered in the form of means and standard deviations in SPSS software. This allowed SPSS to identify heterogeneity between studies using the Q test, *p*-value, and I^2^, which shows the proportion of overall variance caused by heterogeneity across trials (Higgins et al., 2003; Ioannidis, 2008; DerSimonian and Laird, 2015). Hedges’s g was preferred in this study over Cohen’s d because it is usually used for sample sizes with significant differences and eliminates bias by a correction factor (Hedge’s g Statistic, 2017; Lin and Aloe, 2021). I^2^ ≥ 50%, *p* < 0.1 was regarded as substantial heterogeneity between studies, therefore heterogeneity source between them was further analyzed. Moreover, Forest Plots were made using a Forest Plot viewer to see ES distributions and identify outliers. Additionally, 95% confidence intervals were computed for the mean impact size estimate of each study to provide an accuracy metric. Egger’s regression test was utilized by visual examination of funnel plots using the trim-and-fill method to ascertain publication bias (Egger et al., 1997; Duval and Tweedie, 2000). The resultant figure was used to calculate the number of research articles that were overlooked in the meta-analysis and their possible influence on the outcome, as well as to look into possible publication bias.

## 3 Results

### 3.1 Characteristics of included studies

**Figure 1:**
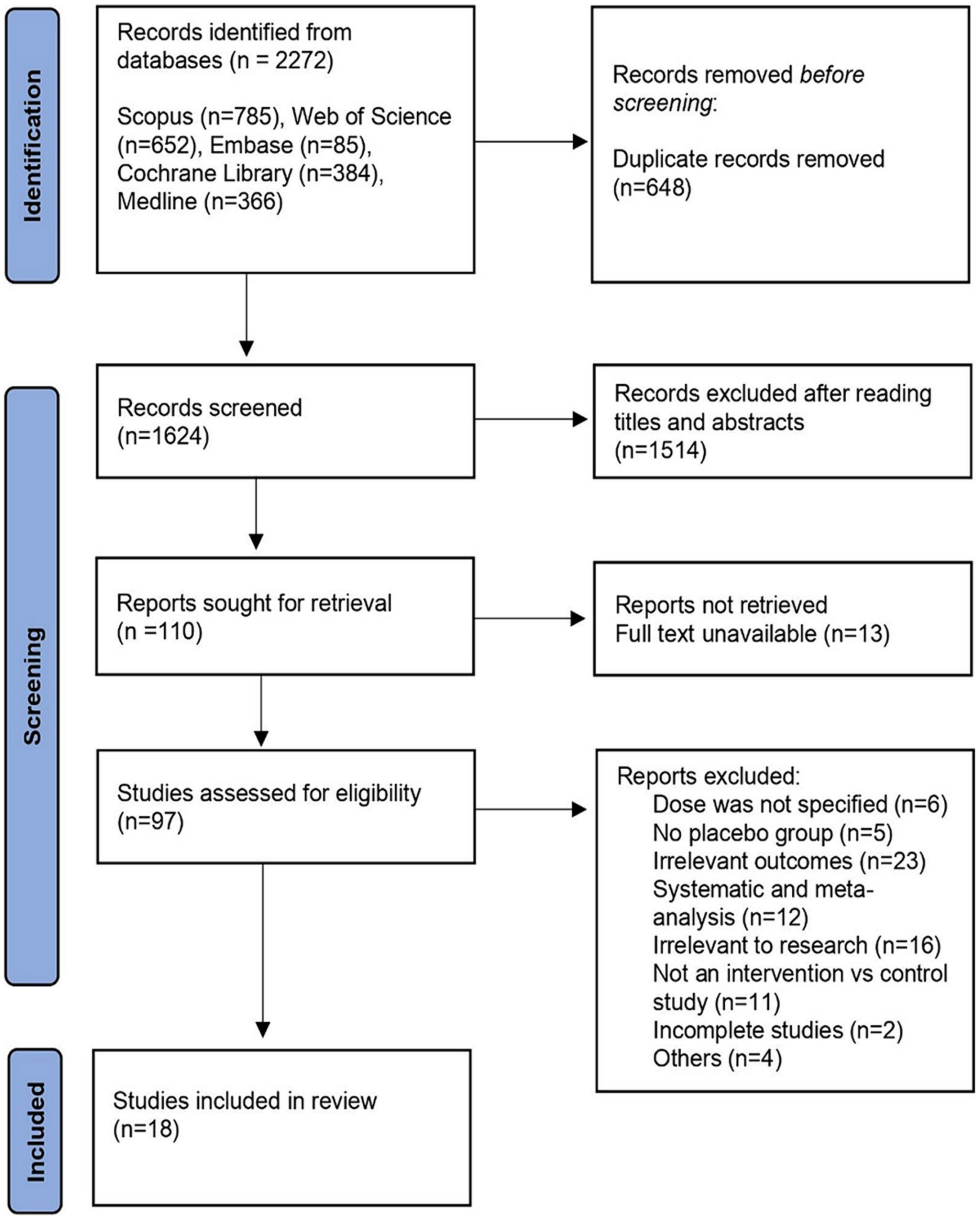
PRISMA flow diagram of the literature selection process.

The search query yielded 652 hits from the Web of Science repository, 785 hits from the Scopus database, and 366 hits from the Medline database. We also searched Embase returning with 85 hits and The Cochrane Library yielding 384 hits. Using the literature search approach, a total of 2,272 articles were found. After the first search, titles and abstracts were manually reviewed, followed by an inclusion/exclusion criterion. Out of these, 18 articles met the requirements for inclusion. A total of 3,770 patients were assigned to a control group (placebo), while 5,989 patients were assigned to a treatment group (donepezil). Figure 1 illustrates the literature screening procedure whereas Table 1 lists the basic characteristics of included articles.

**Table 1:** Basic characteristics of selected studies.

Authors	Year of publication	Region	Clinical group and severity	Mean age (years)	Sample size				Outcome measured in analysis	Duration	Intervention (dose mg/day)		Study design
					Total	Control group	Treatment group				Treatment group	Control group	
							Donepezil (5 mg/d)	Donepezil (10 mg/d)					
Tune et al.	2003	USA	Mild to moderate AD	72.9	28	14	–	14	ADAS-cog	24 weeks	Donepezil (10 mg/d)	Placebo	DBRCT
Black et al.	2003	USA	possible or probable VaD	73.9	603	199	198	206	ADAS-cog, MMSE	24 weeks	Donepezil (5 mg/d, 10 mg/ d)	Placebo	RCT
Wilkinson et al.	2003	Australia	possible or probable VaD	75.0	616	193	208	215	ADAS-cog	24 weeks	Donepezil (5 mg/d, 10 mg/ d)	Placebo	DBRCT
Salloway et al.	2004	USA	Mild cognitive impairment	55–90	270	137	–	132	ADAS-cog	24 weeks	Donepezil (10 mg/d)	Placebo	DBRCT
Holmes et al.	2004	UK	Mild to moderate AD	55	134	55	–	41	MMSE	24 weeks	Donepezil (10 mg/d)	Placebo	DBRCT
Román et al.	2005	Intercontinental	mild to moderate VaD	74.5	1,219	392	406	421	ADAS-cog, MMSE	24 weeks	Donepezil (5 mg/d, 10 mg/ d)	Placebo	DBRCT
Petersen et al.	2005	America, Canada	MCI	55–90	769	259	–	253	ADAS-cog, MMSE	3 years	Donepezil (10 mg/d)	Placebo	DBRCT
Winblad et al.	2006	Sweden	severe AD	≥50	248	120	–	128	MMSE	6 months	Donepezil (10 mg/d)	Placebo	DBRCT
Johannsen et al.	2006	Denmark	Mild to moderate AD	≥50	817	103	–	99	ADAS-cog, MMSE	12–24 weeks	Donepezil (10 mg/d)	Placebo	RCT
Howard et al.	2007	England	AD	<39	272	131	–	128	MMSE	12 weeks	Donepezil (10 mg/d)	Placebo	RCT
Black et al.	2007	Intercontinental	Severe AD	50	343	167	–	176	MMSE	24 weeks	Donepezil (10 mg/d)	Placebo	DBRCT
Dichgans et al.	2008	Germany	CADASIL	54.8	168	82	–	86	ADAS-cog, MMSE	18 weeks	Donepezil (10 mg/d)	Placebo	DBRCT
Moraes et al.	2008	Brazil	mild-to-moderate AD	-	40	12	11	–	ADAS-cog	3 months	Donepezil (5 mg/d)	Placebo	DBRCT
Roman et al.	2010	USA	probable or possible VaD	73.0	1,320	326	648	–	ADAS-cog, MMSE	24 weeks	Donepezil (5 mg/d)	Placebo	RCT
Mori et al.	2012	Japan	DLB	≥50	140	31	32	36	MMSE	12 weeks	Donepezil (5 mg/d, 10 mg/ d)	Placebo	RCT
Ikeda et al.	2015	Japan	mild to moderate or severe AD	≥50	142	44	45	49	MMSE	12 weeks	Donepezil (5 mg/d, 10 mg/ d)	Placebo	RCT
Gault et al.	2015	Intercontinental	Mild–moderate AD	≥65	274	67	–	66	ADAS-cog, MMSE	12 weeks	Donepezil (10 mg/d)	Placebo	RCT
Ridha et al.	2018	UK	AD	61.6	18	10	8	–	MMSE	28 weeks	Donepezil (5 mg/d)	Placebo	DBRCT

CADASIL, Cerebral autosomal dominant arteriopathy with subcortical infarcts and leukoencephalopathy. DBRCT, double-blind randomized controlled trial.

### 3.2 Efficacy outcomes

We conducted the MMSE and ADAS-cog tests to evaluate donepezil’s efficacy in patients with cognitive impairment We examined the impact of two commonly administered doses, i.e., 5 mg and 10 mg, on changes in the cognitive function of the patients in comparison to placebo. To more precisely assess the effectiveness of donepezil, we further divided the studies into additional subgroups.

#### 3.2.1 MMSE

**Figure 2:**
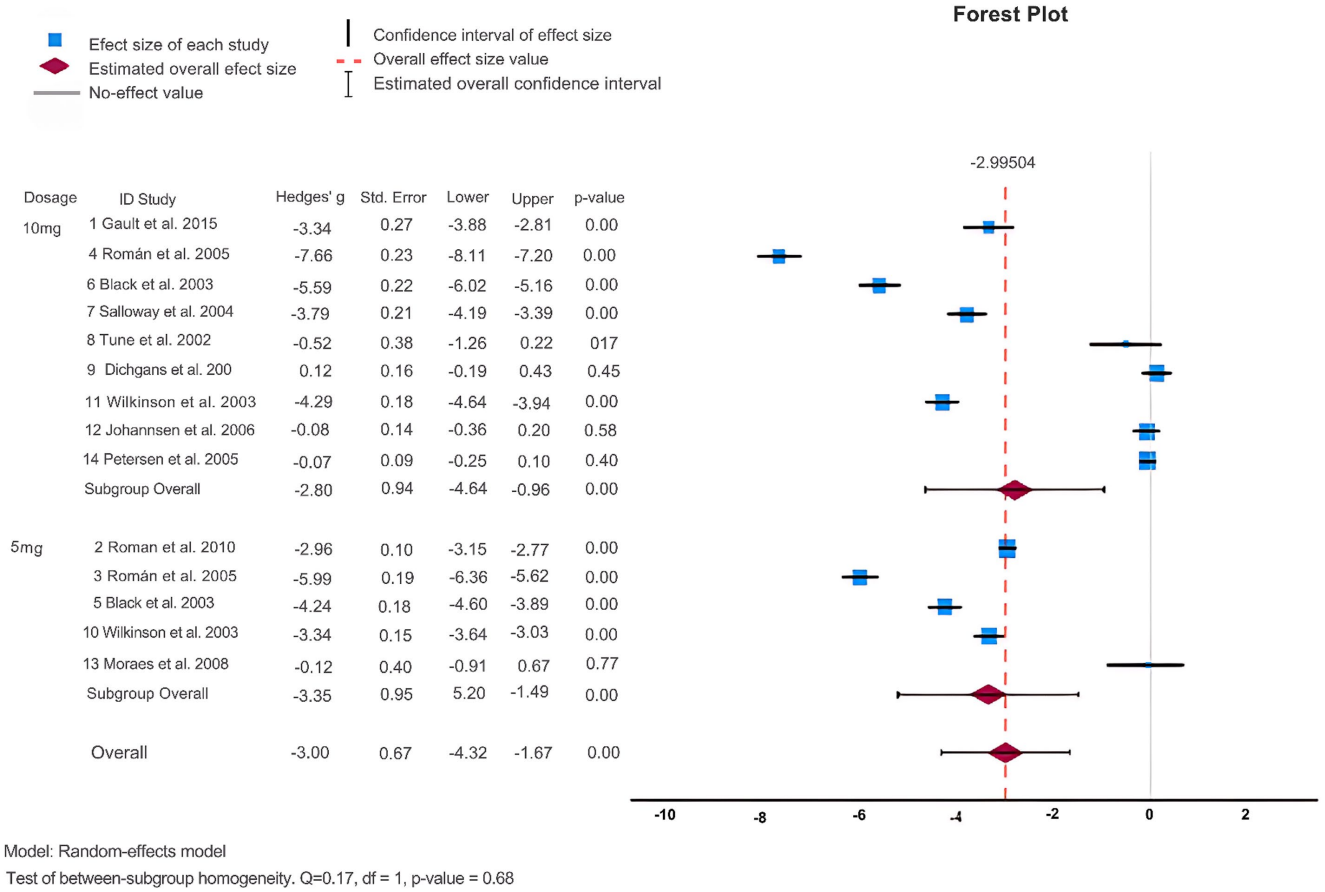
Forest plot of MMSE.

As shown in Table 2 and Figure 2, 14 articles (Black et al., 2003; Holmes et al., 2004; Petersen et al., 2005; Román et al., 2005; Johannsen et al., 2006; Winblad et al., 2006; Black et al., 2007; Howard et al., 2007; Dichgans et al., 2008; Román et al., 2010; Mori et al., 2012; Gault et al., 2015; Ikeda et al., 2015; Ridha et al., 2018) reported the MMSE score change from baseline to endpoint to assess donepezil’s effectiveness in comparison to a placebo. The results of the heterogeneity test indicated a high level of heterogeneity among these studies (I^2^ = 99%) and the Q-test demonstrated statistical significance (Q = 1863.61, *p* < 0.001), indicating that a sizable amount of the variability seems to represent true variation. Consequently, for this meta-analysis, a random effects model was used because the observed heterogeneity did not diminish when we observed different study designs i.e., DBRCT and RCT. The analysis indicated that patients undergoing donepezil treatment significantly improved their MMSE score (Hedges’ g: 2.21, 95%CI: [1.44, 2.98]) favoring intervention over the control group (see Supplementary Figure S4). Furthermore, the results of Egger’s test (*p* = 0.010) showed the absence of publication bias in the included studies.

**Table 2:** Subgroup meta-analysis of efficacy of donepezil against placebo in selected studies.

Outcome	Subgroups	n		Hedges’ g (95%CI)	Heterogeneity (P, Q, I^2^)	Publication bias
		Control group	Treatment group			Egger’s (P)
MMSE	Total (*n* = 14)	1,986	3,026	2.21 (1.44, 2.98)	*p* = <0.001 Q = 1863.614 I^2^ = 99%	0.010
	Region					
	Intercontinental (*n* = 3)	626	1,069	3.39 (1.71, 5.07)	*p* = <0.001 Q = 310.680 I^2^ = 99.1%	0.220
	Europe (*n* = 6)	501	490	2 (0.95, 3.04)	*p* = <0.001 Q = 161.176 I^2^ = 97%	0.194
	North America (*n* = 3)	78	1,305	2.83 (1.71, 5.07)	*p* = <0.001 Q = 867.172 I^2^ = 99.7%	0.548
	Asia (*n* = 2)	75	162	0.68 (0.27, 1.09)	*p* = 0.026 Q = 9.282 I^2^ = 68.8%	0.161
	Dose					
	5 mg/d (*n* = 6)	1,002	1,329	2.09 (0.88,3.30)	*p* = <0.001 Q = 283.485 I^2^ = 98.7%	0.021
	10 mg/d (*n* = 13)	1,650	1,689	2.27 (1.25,3.29)	*p* = <0.001 Q = 1414.977 I^2^ = 99.1%	0.579
	Duration					
	≤ 2 years (*n* = 13)	1,727	2,773	2.33 (1.56, 3.11)	*p* = <0.001 Q = 1161.106 I^2^ = 98.8%	0.003
	>2 years (*n* = 1)	259	253	0.11 (−0.06, 0.29)	*p* = − Q = − I^2^ = −	–
	Clinical groups					
	Alzheimer’s disease (*n* = 8)	697	740	1.71 (0.87, 2.54)	*p* = <0.001 Q = 269.307 I^2^ = 97.2%	0.165
	Vascular dementia (*n* = 3)	917	1879	4.13 (3.14, 5.13)	*p*= <0.001 Q = 236.968 I^2^ = 98.4%	0.613
	Other cognitive impairments (*n* = 3)	372	407	0.88 (0.27, 1.49)	*p* = <0.001 Q = 56.730 I^2^ = 91.8%	0.696
	Study design					
	DBRCT (*n* = 7)	1,085	1,519	2.39 (1.19, 3.59)	*p* = <0.001 Q = 1323.090 I^2^ = 99.2%	0.085
	RCT (*n* = 7)	901	1,507	2.03 (1, 3.05)	*p* = <0.001 Q = 505.403 I^2^ = 98.7%	0.049
ADAS-cog	Total (*n* = 11)	1,784	2,963	−3.00 (−0.25, 0.10)	*p* = <0.001 Q = 2691.172 I^2^ = 99.6%	0.075
	Region					
	Intercontinental (*n* = 2)	459	893	−5.67 (−8.12, −3.21)	*p* = <0.001 Q = 145.923 I^2^ = 98.9%	0.428
	Europe (*n* = 2)	185	185	0.01 (−0.20, 0.22)	*p* = 0.350 Q = 0.874 I^2^ = 0.0%	–
	North America (*n* = 5)	935	1,451	−2.87 (−4.60, −1.14)	*p* = <0.001 Q = 1084.947 I^2^ = 99.5%	0.229
	Oceania (*n* = 1)	193	423	−3.81 (−4.74, −2.87)	*p* = <0.001 Q = 16.235 I^2^ = 93.8%	–
	South America (*n* = 1)	12	11	−0.12 (−0.91, 0.67)	*p* = − Q = − I^2^ = −	–
	Dose					
	5 mg/d (*n* = 5)	1,122	1,471	−3.35 (−5.20, −1.49)	*p* = <0.001 Q = 294.313 I^2^ = 99.4%	0.051
	10 mg/d (*n* = 9)	1,446	1,492	−2.80 (−4.64, −0.96)	*p* = <0.001 Q = 1921.789 I^2^ = 99.6%	0.658
	Duration					
	≤ 2 years (*n* = 10)	1,525	2,710	−3.00 (−4.32, −1.67)	*p* = <0.001 Q = 1806.520 I^2^ = 99.5%	0.032
	>2 years (*n* = 1)	259	253	−0.07 (−0.25, 0.10)	*–*	–
	Clinical group					
	Alzheimer’s disease (*n* = 4)	196	190	−1.02 (−2.57, 0.53)	*p* = <0.001 Q = 116.076 I^2^ = 97.0%	0.690
	Vascular dementia (*n* = 3)	1,110	2,302	−4.86 (−6.08, −3.64)	*p* = <0.001 Q = 548.419 I^2^ = 99%	0.640
	Mild cognitive impairment (*n* = 2)	396	385	−1.93 (−5.57, 1.71)	*p* = <0.001 Q = 274.697 I^2^ = 99.6%	–
	Other cognitive impairments (*n* = 1)	82	86	0.12 (−0.19, 0.43)	*p* = – Q = − I^2^ = −	–
	Study design					
	DBRCT (*n* = 7)	1,089	1746	−2.86 (−4.73, −0.99)	*p* = <0.001 Q = 2039.424 I^2^ = 99.6%	0.150
	RCT (*n* = 4)	695	1,217	−3.24 (−5.03, −1.45)	*p* = <0.001 Q = 589.470 I^2^ = 99.4%	0.798

Subsequently, we performed a subgroup analysis to assess the efficacy of donepezil when administered in doses of 5 mg and 10 mg. We analyzed those patients receiving donepezil 10 mg/day had a significant increase in their MMSE score (Hedges’ g: 2.27, 95%CI: [1.25,3.29]) while 5 mg/day donepezil only slightly managed to increase their MMSE score (Hedges’ g: 2.09, 95%CI: [0.88, 3.30]) as shown in Figure 2. Next, we divided studies into further subgroups for more detailed analysis. When categorized into regions (see Supplementary Figure S1), it was observed that donepezil significantly led to an increase in the MMSE score (Hedges’ g: 3.39, 95%CI: [1.71, 5.07]) in studies conducted intercontinental. In terms of study duration, studies of more than 2 years showed an increase in MMSE score, while no significant difference was observed in studies of less than 2 years (Hedges’ g: 2.33, 95% CI: [1.56, 3.11], I^2^ = 98.8%) indicating that study duration might be a source of heterogeneity (see Supplementary Figure S2). Furthermore, when analysis was performed in subgroups based on clinical groups, patients with VaD demonstrated an increase in MMSE score (Hedges’ g: 4.13, 95%CI: [3.14, 5.13], I^2^ = 98.4%) whereas other clinical groups did not exhibit significant difference (see Supplementary Figure S3).

#### 3.2.2 ADAS-cog

**Figure 3:**
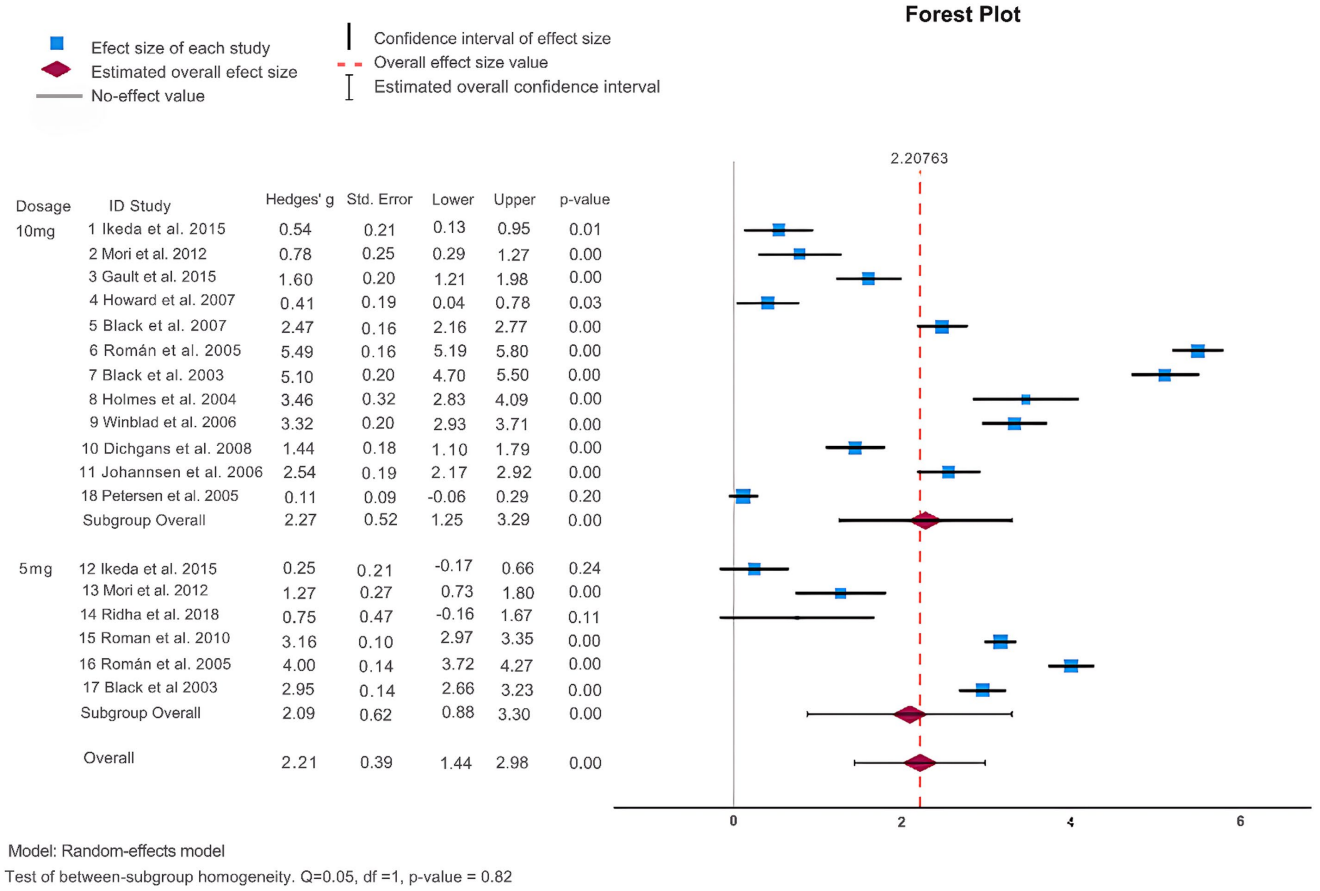
Forest plot of ADAS-cog.

Additionally, ADAS-cog scores were also examined. As shown in Figure 3 and Table 2, 11 articles (Black et al., 2003; Holmes et al., 2004; Salloway et al., 2004; Petersen et al., 2005; Román et al., 2005; Johannsen et al., 2006; Dichgans et al., 2008; Román et al., 2010;^29,82^; Wilkinson et al., 2003) employed ADAS-cog scores to assess the effectiveness of donepezil in treating dementia. The heterogeneity test revealed a high degree of heterogeneity among the studies (*p* = <0.001, Q = 2691.172, I^2^ = 99.6%). When the random effects model was used for the meta-analysis, Egger’s test (*p* = 0.075) revealed that publication bias was absent. The findings demonstrated that donepezil tended to lower ADAS-cog scores and enhance cognitive function in patients as compared to the control group. The studies were divided into subgroups, and ADAS-cog scores were analyzed. When categorized into regions, donepezil led to an increase in ADAS-cog scores (Hedges’ g: –5.67, 95%CI: [−8.12, −3.21]) only in studies with subjects from intercontinental locations (see Supplementary Figure S5). The heterogeneity test indicated a high degree of heterogeneity (*p* = <0.001, Q = 145.923, I^2^ = 98.9%) in these subgroups. When distinguished by the dose of donepezil (5 mg vs. 10 mg), statistically there was no substantial difference in ADAS-cog scores (Hedges’ g: -2.80, 95%CI: [−4.64, −0.96]) as shown in Figure 3. When different clinical groups were considered, there was a substantial increase in heterogeneity (*p* = <0.001, Q = 548.419, I^2^ = 99%) among VaD patients compared to other clinical groups (see Supplementary Figure S7). Similarly, the division into RCT and DBRCT groups did not reveal a significant difference in the heterogeneity test (see Supplementary Figure S8).

### 3.3 Safety outcomes

**Figure 4:**
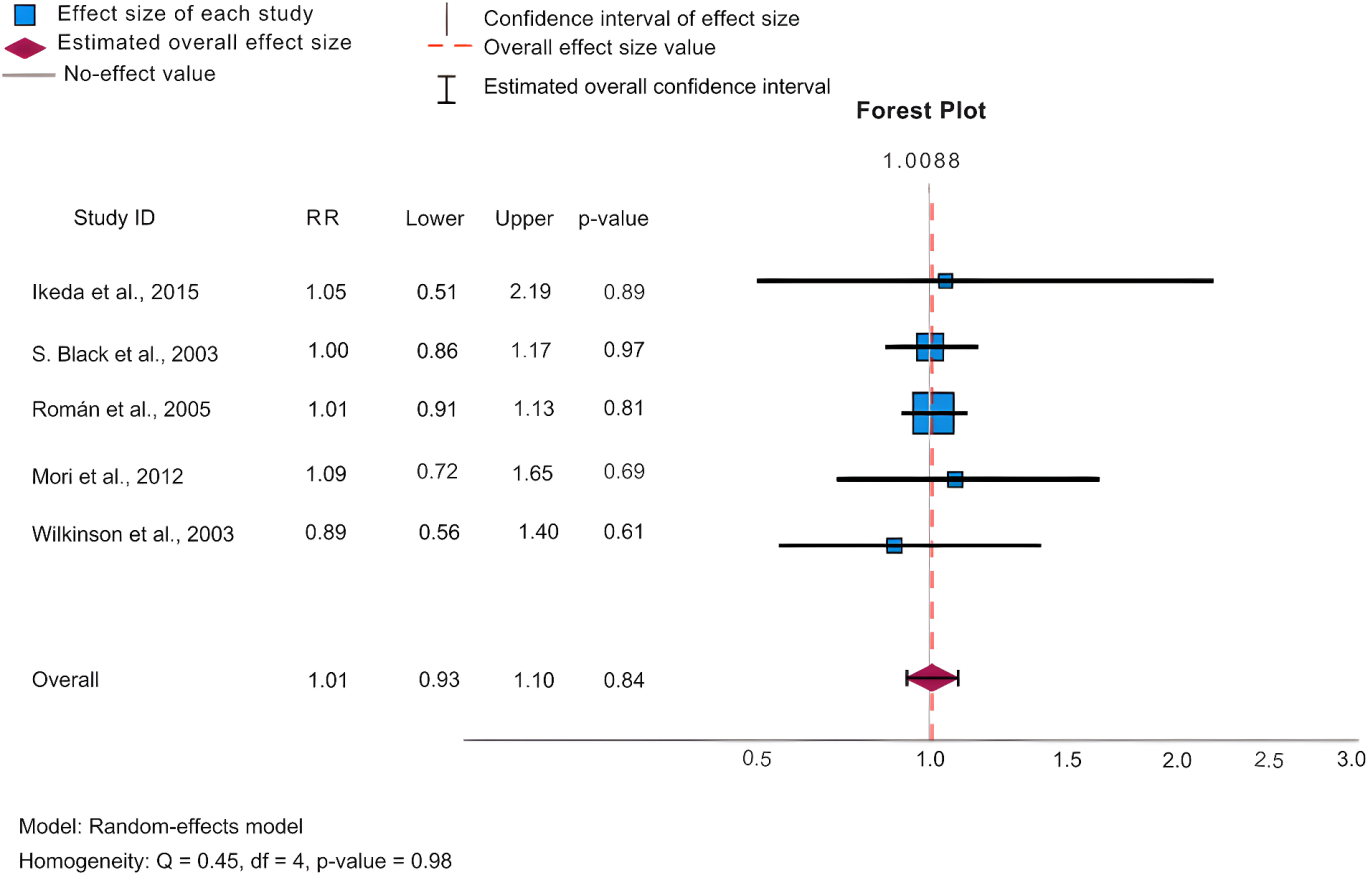
Forest plot of ADRs at 5 mg/day.

**Figure 5:**
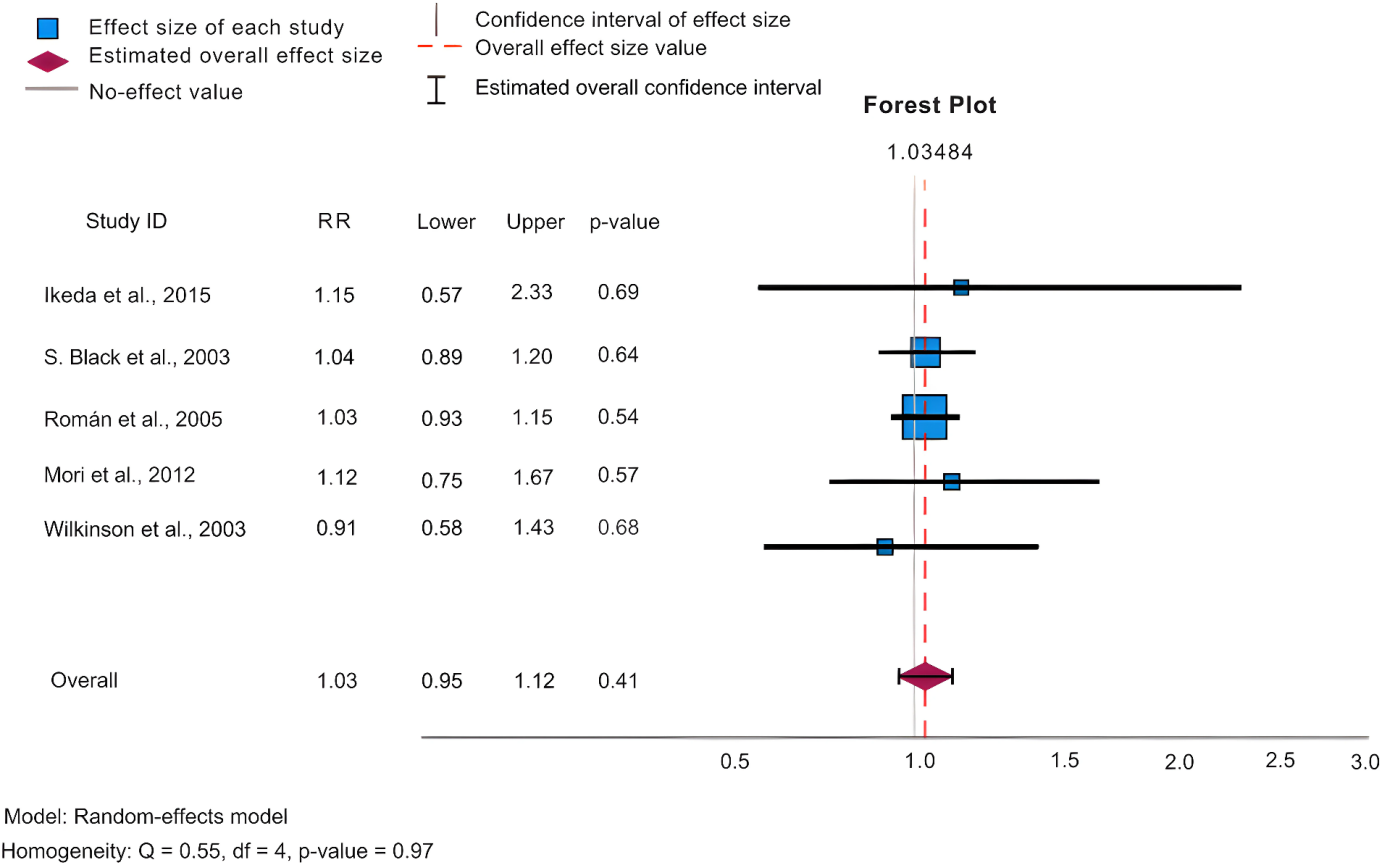
Forest plot of ADRs at 10 mg/day.

In our analysis, we conducted statistics of common adverse effects observed during the trial, with 5 articles (Black et al., 2003; Wilkinson et al., 2003; Román et al., 2005; Mori et al., 2012; Ikeda et al., 2015) contributing to the analysis. As depicted in Figure 4 for 5 mg/day, Figure 5 for 10 mg/day, and Table 3. The heterogeneity test for the 10 mg/day dosage indicated significant variation among the studies (*p* = <0.001, Q = 715.425, I2 = 98.9%), using the random effects model for the meta-analysis. Our findings revealed that the likelihood of experiencing adverse reactions with 10 mg/day of donepezil (RR = 1.07, 95%CI = 1.03, 1.11) was higher compared to 5 mg/day (RR = 1.03, 95%CI = 0.99, 1.07). The Egger’s test (*p* = 0.219) in the case of 5 mg/day, and (*p* = 0.664) in the case of 10 mg/day suggested no publication bias. Furthermore, we conducted thorough observations of adverse reactions during the trials, selecting adverse effects based on their prevalence and notable differences between the 5 mg/day and 10 mg/day doses. As presented in Table 3, the findings revealed that the 10 mg/day treatment group had a higher risk for adverse events such as nausea/vomiting, diarrhea, anorexia, hypertension, and abnormal dreams. The differences were statistically significant across most adverse events, except for nausea where the 5 mg/day group showed slightly higher results (RR = 0.23, CI95% = 0.20, 0.27).

**Table 3:** Meta-analysis of safety outcomes.

Outcome	Dose (n)	T event (n)	T sample (n)	C event (n)	C sample (n)	RR (95%CI)	Heterogeneity (P, Q, I^2^)	Publication bias
								Egger’s (P)
Total ADRs (*n* = 5)	5 mg/d	767	892	721	864	1.03 (0.99, 1.07)	*p* = <0.001, Q = 715.425, I^2^ = 98.9%	0.219
	10 mg/d	830	928			1.07 (1.03, 1.11)	*p* = 0.994, Q = 0.741, I^2^ = 0.0%	0.664
Nausea/Vomiting (*n* = 5)	5 mg/d	174	892	721	864	0.23 (0.20, 0.27)	*p* = 0.617, Q = 2.656, I^2^ = 0.0%	−0.574
	10 mg/d	170	928			0.22 (0.19, 0.25)	*p* = 0.483, Q = 3.466, I^2^ = 20.8%	0.104
Diarrhea (*n* = 3)	5 mg/d	63	439	367	426	0.17 (0.13, 0.21)	*p* = 0.542, Q = 1.225, I^2^ = 0.0%	0.499
	10 mg/d	73	458			0.19 (0.15, 0.23)	*p* = 0.530, Q = 1.271, I^2^ = 0.0%	0.334
Anorexia (*n* = 5)	5 mg/d	57	892	721	864	0.08 (0.06, 0.10)	*p* = 0.624, Q = 1.758, I^2^ = 0.0%	0.426
	10 mg/d	76	928			0.10 (0.08, 0.12)	*p* = 0.528, Q = 3.183, I^2^ = 0.0%	0.319
Hypertension (*n* = 3)	5 mg/d	42	812	686	784	0.06 (0.04, 0.08)	*p* = 0.834, Q = 0.363, I^2^ = 0.0%	0.898
	10 mg/d	48	842			0.07 (0.05, 0.09)	*p* = <0.883, Q = 0.248, I^2^ = 0.0%	0.954
Abnormal dreams (*n* = 3)	5 mg/d	22	812	686	784	0.03 (0.02, 0.05)	*p* = 0.842, Q = 0.345, I^2^ = 0.0%	0.959
	10 mg/d	48	842			0.07 (0.05, 0.09)	*p* = 0.622, Q = 0.951, I^2^ = 0.0%	0.743

Tevent, Number of events in the treatment group; Tsample, Sample size of treatment group; Cevent, Number of events in the control group; Csample, Sample size of the control group; ADRs, Adverse drug reactions.

## 4 Discussion

This study was intended to ascertain the effectiveness of donepezil in cognitive impairments in doses. This meta-analysis of placebo-controlled trials revealed substantial differences between the donepezil and placebo groups based on MMSE (14 studies, 5,012 participants) and for the ADAS-Cog analysis (11 studies, 4,747 participants). A combination of comparative trials evaluating donepezil’s dose–response at 5 mg/d and 10 mg/d suggested that a higher dosage i.e., 10 mg/day of donepezil is favorable for maintaining or slowing down the progress of cognitive impairment in patients with any stage of dementia. All the patients with AD, Vad, and other cognitive impairments showed significant improvement in MMSE scores and a moderate improvement in ADAS-cog scores and thus these enhancements in cognition appear to have a positive effect on the functioning of the patients with dementia.

Our findings are similar to previous done meta-analyses studies including control vs. treatment group results; however, we did not just limit our study to only one factor. We divided our study into subgroups of duration, dose, region, clinical group, and study design to get more precise results of donepezil efficacy in patients. Previous meta-analysis has been done with either only a specific stage of dementia or its specific type, however, we selected all available studies with dementia i.e., AD, VaD, MCI, CADASIL, and DLB to expand our research, making our conclusions more reliable.

Our main focus throughout the studies was the dose effectiveness of donepezil measured by MMSE and ADAS-Cog scores. Through our results, we found out that 10 mg/day of donepezil was more effective than 5 mg/day and this is concurrent with the results of studies done by Whitehead et al. (2004), Birks and Harvey (2018), and Govind (2020) in case of AD. However, if compared to other treatment strategies both 5 mg and 10 mg donepezil are the most effective strategies for treatment according to results in studies (Kobayashi et al., 2016; Blanco-Silvente et al., 2017; Zhang et al., 2020) in the case of AD. In the case of severe AD, MMSE scores were improved at 10 mg/day of donepezil and this result is also shown by Adlimoghaddam et al. (2018).

Our study also revealed that in the case of MCI, giving 10 mg/day of donepezil showed significant improvement in MMSE scores but no substantial progress in ADAS-cog score and this result was in line with a study done by Zhang X. et al. (2022). Whereas in the case of VaD, our results showed improved ADAS-cog scores, which are in line with the results of Chen et al. (2016), however, our results showed moderate improvement in MMSE scores too which was not concurrent in the latter but was in line with the studies done by Shi et al. (2022) at dose of 10 mg. We could not conclude decisively in the case of DLB and CADSIL due to limited available research as well as very small sample sizes. However, our limited results showed no statistically significant improvement on cognitive scales. ADAS-cog scores were moderately improved, though MMSE scores did not show much improvement.

When subgroups were distinguished by geography, patients from Europe showed significantly enhanced MMSE scores. The subjects’ Asian origin rendered this distinction insignificant. However, studies performed across multiple continents and in North America alone showed moderate improvement as well. Furthermore, subgroup analyses based on clinical groups, dose, region, duration, and type of intervention could not mitigate the high level of heterogeneity among studies. This indicated that individual studies were highly likely to be the source of heterogeneity. Furthermore, one of the possible reasons for heterogeneity among 5 mg and 10 mg studies can be due to the different stages of severity in each study.

Moreover, as the results of this study and previous trials included in this review, are evidence of the fact that donepezil 10 mg/day is more efficacious than donepezil 5 mg/day, the adverse drug reactions (ADRs) of donepezil 10 mg/day, however, are more than donepezil 5 mg/day. One study suggests that ADRs associated with donepezil 10 mg/day are thought to be connected to a quick escalation up to 10 mg/day within 1 week of commencing medication, which was the usual procedure in the donepezil pivotal trials (Doody et al., 2008). However, if we compare 10 mg with higher doses of donepezil, many recent studies show that higher doses, i.e., 15 mg/day up to 23 mg/day of donepezil have more serious side effects and poor safety profiles besides the fast improvement of cognitive symptoms (Study Results, 2007; Hong et al., 2019; jia et al., 2020; Study Results, 2021; Mori et al., 2024a,b). When discussing ADRs, similar studies and a study by Birks and Harvey (2018) suggest that 10 mg/day of donepezil shows mild to moderate ADRs commonly nausea, vomiting, diarrhea, and dizziness as compared to higher doses responsible for ADRs like bradycardia, and urinary incontinence. Given all of these findings, donepezil 10 mg/day appears to be an optimal choice for reducing cognitive problems compared to donepezil 5 mg/day and better tolerability and safety profile compared to higher doses. Although to overcome ADRs of 10 mg/day of donepezil, studies suggest that 10 mg/day of donepezil in combination therapy with agents like memantine shows similar improvement in cognitive symptoms with a better safety profile and tolerable ADRs in contrast to monotherapy of donepezil at 10 mg and higher doses (Rong et al., 2021; Knorz and Quante, 2022).

### 4.1 Study strengths

This meta-analysis attempted to collate all the published RCT studies on dementia conducted according to inclusion criteria. Previous meta-analyses have opted to limit their analysis to a certain severity range, such as mild to moderate or severe. This was done with the idea that the severity of the disease may have an impact on the medication’s effectiveness. Regardless of the length of the trials or the severity of dementia in the patients, our research found very little evidence that the treatment effects varied between studies. This would corroborate our choice to incorporate all research, regardless of the degree of severity.

### 4.2 Study limitations

The current study has some limitations, which should be taken into account when interpreting its results. This study was intended to determine the efficacy of donepezil at standard doses; therefore, this meta-analysis excluded the studies that were conducted at higher doses of donepezil, i.e., more than 10 mg. The precision of results for dementia types other than AD and VaD was compromised. This might be due to a limited number of studies that fulfilled the inclusion criteria as well as the small size of these studies. Additionally, the present meta-analysis did not encompass unpublished research or data, which could not be made available even after the requests to the authors.

## 5 Conclusion

This meta-analysis suggests that as compared to placebo, commonly prescribed cholinesterase inhibitors, donepezil (5 and 10 mg/day) is effective for symptomatic treatment for patients with dementia. All studies included in this meta-analysis showed a positive impact of donepezil on stabilizing and delaying the development of cognitive impairment, with some studies showing statistical significance over others. Donepezil at both doses is efficacious, however, 10 mg/day at 24 weeks is more likely to execute the utmost gain.

## Data availability statement

The original contributions presented in the study are included in the article/Supplementary material, further inquiries can be directed to the corresponding author.

## Author contributions

MS: Writing – original draft, Formal analysis, Methodology, Software. MA: Writing – review & editing, Validation.
